# Patient Participation in Monitoring Potential Adverse Drug Events

**DOI:** 10.1055/a-2641-0265

**Published:** 2025-11-14

**Authors:** Kerstin Jorsäter Blomgren, Johan Fastbom

**Affiliations:** 1 School of Health, Care and Social Welfare, Mälardalen University, Eskilstuna, Sweden; 2 Aging Research Center, Karolinska Institutet and Stockholm University, Stockholm, Sweden

**Keywords:** clinical decision support, adverse drug event, self-reporting, medication review

## Abstract

**Background:**

Clinical decision support systems (CDSS) have been suggested to be helpful in detecting and preventing drug-related problems such as adverse drug events (ADEs). However, patient participation systems monitoring self-reported data, such as symptoms, are still sparsely described in the literature.

**Objectives:**

This study aimed to investigate if the use of a patient participating CDSS (PCDSS) can facilitate early detection of ADEs, thereby contributing to safer drug treatment in older adults.

**Methods:**

A 1-year prospective observational study of elderly patients using a free web-based PCDSS to register symptoms over time at home. Initially, the PCDSS analyzed the extent and quality of the patient's drug use, based on a Swedish national set of criteria, and assessed drug-related symptoms using a standardized scale (PHASE-20). Thereafter, the patients recorded symptoms at home for 1 year—the first 6 months in free text, the second 6 months selecting from 19 predefined symptoms. The PCDSS signaled when symptoms were registered on three occasions in a 3-week period. The patient was then asked to contact his/her nurse at the healthcare center (HCC) for assessment of the symptoms and decisions on further contacts with the nurse or doctor. We analyzed the extent of signals generated, accompanying contacts, and associated medication reviews and adjustments.

**Results:**

The 48 study participants registered 1,275 symptoms during the monitoring period, 61% by women. The PCDSS generated a total of 171 signals, of which 58% from women. Seventy-one percent (121) occurred under the first registration (free text) period. Of all signals, 44% (75) led to activities at the HCC, of which 48% (36) were physician contacts. In total, they contributed to medication reviews in 42% (15) and medication adjustments in 64% (23), with a total of 33 adjustments.

**Conclusion:**

Patient participation by self-reporting symptoms via a PCDSS can contribute to safer drug use.

## Background and Significance

In Sweden, as in many other countries, drug use in older adults has increased significantly over the last decades. 1 Extensive drug use in older adults increases the risk of drug-related problems; due to age- and disease-related physiological changes that lead to an increased sensitivity to drugs, 2 as well as the consequences of polypharmacy, including higher risk of nonadherence, drug–drug interactions (DDI), and adverse reactions (ADR). 3

Drug use in older people is becoming more complex and, thus, requires careful and regular evaluation of the risks in relation to the benefits. Drug-related problems, including ADRs and DDI, are the underlying cause of up to 30% of the hospitalizations of elderly patients, creating unnecessary suffering and increased healthcare costs. 4 5 6 A report from the Swedish National Board of Health and Welfare 7 points out that about one in ten hospitalizations among older adults is caused by ADRs, at least half of which are estimated to be preventable.

The importance of patients' participation in their own healthcare has been extensively discussed over the last years. Several studies have described the value and benefits of cooperation between caregivers and patients to ensure high-quality care. 8 9 10 11 Improving medication safety is the overall goal. However, most existing studies on this subject focus on healthcare-oriented measures such as prevalence of potential DDI and drug-related hospitalizations, 12 whereas patient-reported measures, such as drug-related symptoms and signs, are sparsely described. 13

Clinical decision support systems (CDSS) have been shown in several studies to have the potential to reduce inappropriate medication use in older adults. However, there are also data showing no significant or questionable effects, primarily regarding the prevention of adverse drug events (ADEs). 14 15 16 17 18 19 Concerns have been raised about the efficiency of these systems, as well as their limitations, such as generating false alerts and not contextually relevant information, which have affected their implementation in clinical practice. 20 21 However, recent findings demonstrate their effectiveness. 22 23 Further, one study describes the development of an advanced CDSS as being highly effective in preventing ADEs at the prescribing stage. 24 Moreover, several systematic overviews highlight both the benefits of using CDSS and the difficulties and challenges to succeed. 25 26 27 28

The aim of this study was to try a model for detecting early signs and symptoms of potential ADEs among older patients living in their own homes, where the patients can directly report their symptoms over time, using a patient-centered web-based CDSS (PCDSS). A nurse at the healthcare center (HCC) acts as a filter for any further action, for example, a nurse or physician visit or a medication review, by providing the initial contact with the patient and reviewing the patient's reported symptoms.

## Objectives

To evaluate whether the use of a PCDSS can facilitate the early detection of ADEs at the individual level and thereby contribute to safer drug treatment.

## Methods

### Setting

This study was performed at two regionally owned HCCs located in the middle part of Sweden. Purposive sampling was used for recruiting the HCCs, and both had previous experience with using a CDSS. One of the HCCs was located in a city with approximately 150,000 inhabitants, of which 13,739 patients were listed at the HCC at the start of the study, and the other one was in a sparsely populated area of 4,300 inhabitants, with 2,500 patients listed.

### Design and Details of the Clinical Decision Support Systems

“miniQ” (QP Medtech International AB; *https://qualitypharma.se* ) is a web-based CDSS for prescribing and medication reviews in older adults. The system consists of three modules: one for the physician/nurse, one for a consultant expert (pharmacist and clinical pharmacologist), and one for the patient/relative. The patient module, SeniorminiQ, was developed in collaboration with one of the larger Swedish pensioners' organizations. It was freely available on the internet, providing the possibility for an elderly person, or his/her relatives, to enter information about his/her current drug use and symptoms. The CDSS analyzes the quality of the drug use based on a set of criteria, in this case the national indicators issued from the Swedish National Board of Health and Welfare 29 —including, for example, inappropriate drugs, duplicate medication and potential DDI, as well as potential ADRs based on cross referencing the current drug use with an assessment of the patient's symptoms using the PHASE-20 rating scale, developed for the identification of possible drug-related symptoms among elderly. 30

From this analysis, a basis for discussion is generated with questions about the quality of drug use and potential ADRs. A printout of this may be brought to the doctor's appointment, supporting the discussion with the physician. This empowers the patient by providing him/her with adequate questions to ask about the benefits and risks of the current medication. The data entered in SeniorminiQ may also be imported for further analysis into the physician's/nurse's module miniQ, providing the basis for a medication review.

For this study, we developed a “vigilance module” (VM; for the patient referred to as “diary”) which was added to the existing SeniorminiQ, for regular monitoring of symptoms by the patient during the study period.

### Patient Sample and Procedure

Potential study patients were sought out through a search engine (Medrave4 Primärvård) connected to the medical record system, where inclusion criteria concerning age, number of drugs used, and HCC affiliation were defined. A total of 397 (161 men, 236 women; Table 1 ) patients were selected, and an information letter with a request about interest to participate was sent to each of them in a consecutive manner.

**Table 1:** Reasons for exclusion after screening and characteristics of the final study population

Screened persons, reasons for exclusion	Men; *n* = 161	Women; *n* = 236	Total; *n* = 397
No computer access, *n* (%)	40 (25)	78 (33)	118 (30)
Not interested in participating, *n* (%)	41 (26)	48 (20)	89 (22)
No contact, *n* (%)	27 (17)	45 (19)	72 (18)
Language difficulties, *n* (%)	1 (0.6)	3 (1.3)	4 (1.0)
Not able to manage; due to severe dementia or other disease, being “too old,” need of municipal home care, etc., *n* (%)	18 (11)	15 (6.4)	33 (8.3)
Use of less than 5 drugs, *n* (%)	2 (1.3)	10 (4.2)	12 (3.0)
Incorrectly selected for screening, or change of healthcare center, *n* (%)	3 (1.8)	11 (4.7)	14 (3.5)
**Study population, characteristics**	Men; *n* = 25	Women; *n* = 23	Total; *n* = 48
Age, mean (range)	80.4 (75–87)	80.7 (76–90)	80.5 (75–90)
Medications at baseline, mean (range)	8.6 (5–15)	8.6 (4–14)	8.6 (4–15)
Symptoms recorded as moderate or major in PHASE 20, n (mean)	105 (4.2)	119 (5.2)	224 (4.7)

Patients were considered eligible for participation if they were: aged 75 years or over, prescribed five or more drugs, living in their own home without home healthcare, and listed to any of the participating HCCs. Exclusion criteria were no access to a computer or not managing the Swedish language.

A total of 55 patients (29 men and 26 women) were finally enrolled in the study ( Table 1 ). Each patient was called to a first meeting at the HCC, where detailed information about the study process was provided by the coordinator, and an “informed consent” was sought. If the patient agreed to participate, information about his/her current medication, as well as a symptom assessment according to PHASE 20, 30 was entered into SeniorminiQ. A basis for discussion based on an analysis of this information, regarding quality of drug use and potential ADRs, was then printed out and given to the patient.

Each patient received a personal code for access to the PCDSS and was asked to register any experienced symptoms twice a week in the VM (diary). In the first 6 months of the study period, the patients registered freely in a text field (spontaneous registrations), while during the second 6 months, they were presented with a list of 19 different symptoms, of which they could select one or more (predefined registrations). The suggested symptoms were based on typical prodromal symptoms of conditions related to the ADRs most frequently causing hospitalizations among elderly people ( Table 2 ). 7 The idea was that both methods had their advantages. The former gives the patients the freedom to freely record their symptoms, both in terms of scope and wording, while the latter might make it easier for them to interpret and report a symptom as a possible ADE.

**Table 2:** List of symptoms to choose from for the predefined registrations

Fatigue
Dizziness
Feeling faint
Unsteady
Muscle weakness
Fall
Worry/anxiety
Cloudiness/confusion
Headache
Pallor
Sweat
Palpitations or low heart rate
Swollen
Short of breath
Poor appetite
Nausea or vomiting
Stomach-ache
Diarrhea
Dark stools

**Fig. 1:**
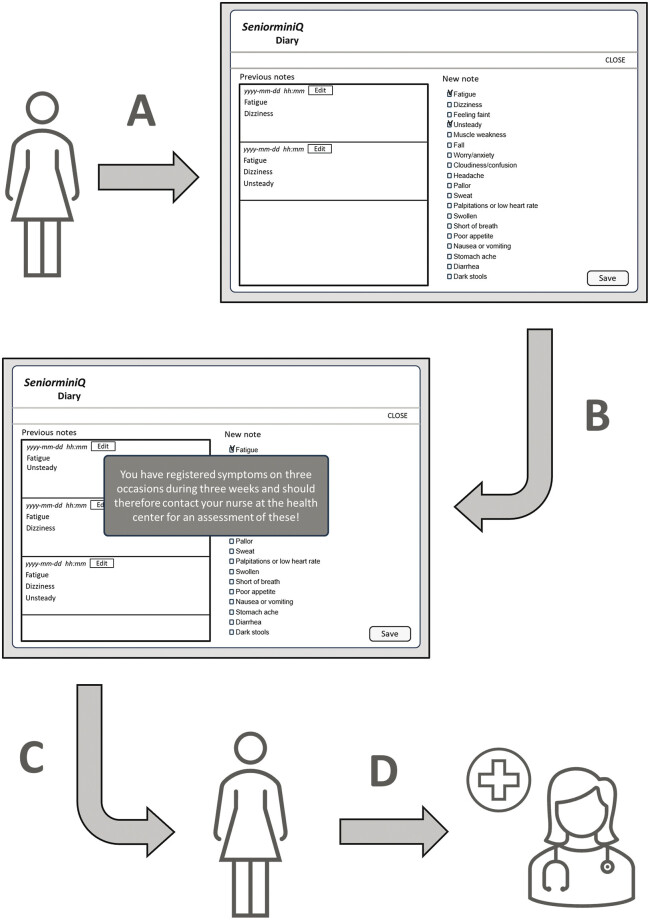
Description of the process of self-reporting of symptoms to the HCC, via the vigilance module. ( **A** ) The patient registers any experienced symptoms twice a week in the web-based vigilance module, either as free text (first 6 months) or by choosing among 19 predefined symptoms (second 6 months). ( **B** ) When at least three registrations have been made during a 3-week period, the system signals that the patient should contact the nurse responsible at the HCC for an assessment. ( **C** ) The patient recognizes the alert and ( **D** ) calls the nurse, who logs in to the system and goes through the registered symptoms, to assess whether any of them may be related to ADEs or, for other reasons, should be further investigated.

The VM was programmed to signal when symptom recordings were made on three occasions over 3 weeks. When this occurred, the patient received a request via the system to contact her/his responsible nurse for assessment of the registered symptoms, after which the nurse decided whether they warranted contact with a nurse or physician ( Fig. 1 ).

The documentation from the VM and the basis for discussion from the PCDSS were proposed to form the basis for the physician visit and a medication review. The result of these actions, in terms of changes in drug prescriptions and other decisions, was documented in the patient's medical record.

### Quality Assurance and Control

At study start, the HCC received information about and training in the procedure from the study coordinator. Coordinating activities, including regular visits to HCC and, when necessary, telephone contacts, were performed throughout the study period. Further, reviews of the recordings in the VM were regularly made by the study coordinator. The study patients, as well as the study nurses, were able to contact the coordinator at any time during the study period.

## Results

Of a total of 397 potential study participants, 30% stated no access to a computer or tablet as a reason for not being able to participate. Twenty-two percent were not interested in participating, without stating any reason. For eighteen percent of the potential study subjects, we were unable to establish any contact at all. For details about the excluded patients, see Table 1 . Finally, 55 patients were enrolled. Of these, another seven patients dropped out during the study period. Thus, the final study population comprised 48 patients.

### Basic Characteristics of the Included Patients

Among the finally included and monitored study subjects, the mean age was similar in men and women, 80.4 and 80.7, respectively. The mean number of drugs used per person was also similar, 8.6 in men and 8.6 in women. In the initial symptoms assessment based on PHASE-20, 30 the participants reported on average 4.7 symptoms (men: 4.2; women: 5.2), out of the 20 listed in the form, creating moderate or major discomfort ( Table 1 ).

### Measures of Polypharmacy and Prescribing Quality at Baseline

Polypharmacy (use of ≥5 drugs) was seen in 46 (96%) and excessive polypharmacy (use of ≥10 drugs) in 16 (33%) of the participants. Use of inappropriate drugs according to the Swedish criteria, 29 including long-acting benzodiazepines, anticholinergic drugs, tramadol, and propiomazine, was relatively uncommon ( *n* = 4; 8%), whereas DDI were fairly frequent. DDIs of class C (the drug combination can lead to changed effects or adverse events, but can be handled with individual dosage adjustments) were seen in 16 (33%) and class D (the combination should be avoided) in three (6%) patients. Nonsteroidal anti-inflammatory drugs (NSAIDs) were used by eight (17%) patients.

### Symptoms Reported at Baseline

Tired/exhausted and dry mouth, both reported by 24 (50%) participants, were the most frequent symptoms assessed as giving moderate or major discomfort, followed by swollen legs/ankles ( *n* = 19; 40%), short of breath ( *n* = 19, 40%) and dizzy/unsteady/high risk of falls ( *n* = 18; 38%; Table 3 ).

**Table 3:** Symptoms reported to give moderate or major discomfort, in assessments (PHASE 20) at baseline, *n* (%)

Symptoms	Men; *n* = 25	Women; *n* = 23	Total; *n* = 48
Tired/exhausted	13 (52)	11 (48)	24 (50)
Dry mouth	10 (40)	14 (61)	24 (50)
Swollen legs/ankles	11 (44)	8 (35)	19 (40)
Short of breath	9 (36)	10 (43)	19 (40)
Dizzy/unsteady/high risk of falls	8 (32)	10 (43)	18 (38)
Other symptoms (e.g., pain)	9 (36)	8 (35)	17 (35)
Frequent urination/incontinence of urine	8 (32)	8 (35)	16 (33)
Poor sleep pattern/nightmares	3 (12)	9 (39)	12 (25)
Itching/rash	6 (24)	4 (17)	10 (21)
Abdominal pain/chest pain	2 (8)	7 (30)	9 (19)
Forgetful	7 (28)	1 (4)	8 (17)
Constipation	3 (12)	5 (22)	8 (17)
Palpitations (rapid/irregular heartbeat)	3 (12)	5 (22)	8 (17)
Irritable	4 (16)	2 (9)	6 (13)
Worried/anxious	3 (12)	2 (9)	5 (10)
Nausea/vomiting	2 (8)	2 (9)	4 (8)
Low mood	2 (8)	2 (9)	4 (8)
Poor appetite	2 (8)	1 (4)	3 (6)
Diarrhea	0 (0)	1 (4)	1 (2)
Headache	0 (0)	0 (0)	0 (0)

### Symptoms Reported in the Vigilance Module during the Study

The 48 study participants made a total of 689 symptom registrations, that is, an average of 14 (women: 21; men: 8) per person, during the monitoring period. Seventy percent of these registrations were made by women. Each patient made 1 to 94 registrations (women: 2–94; men: 1–65). Half of the patients made ≥5 registrations. On average, 1.7 symptoms were recorded per registration (women: 1.9; men: 1.4).

A total of 1,275 symptoms (an average of 26 per person; men: 20; women: 34) were recorded; 61% by women. The spontaneous registrations accounted for 70% of the symptoms. Among the recorded symptoms, pain dominated, with 243 (19%) of all registrations. This was recorded predominantly by women (73%). The second most recorded symptom was tiredness (14%), dominated by women, followed by unsteadiness (12%) and respiratory symptoms (9%), mostly in men ( Table 4 ).

**Table 4:** Number of symptoms registered and the 10 most registered symptoms during the study period, sorted by decreasing order of frequency in the study population

Symptoms	Men; *n* = 25	Women; *n* = 23	Total; *n* = 48
Spontaneous registration, *n*	385	503	888
Predefined registration, *n*	116	271	387
Total, *n*	501	774	1,275
Symptoms, *n* (% of total)			
Pain	66 (13)	177 (23)	243 (19)
Fatigue	81 (16)	150 (19)	231 (18)
Unsteadiness	82 (16)	78 (10)	160 (12)
Respiratory symptoms	91 (18)	25 (3.2)	116 (9.1)
Gastrointestinal symptoms	17 (3.4)	92 (12)	103 (8.1)
Dizziness	28 (5.6)	33 (4.3)	61 (4.8)
Muscle weakness	15 (3.0)	43 (5.6)	58 (4.5)
Swelling	33 (6.6)	18 (2.3)	50 (3.9)
Headache	8 (1.6)	42 (5.4)	50 (3.9)

### Signals Generated from the Vigilance Module and Related Activities during the Study Period

The VM generated a total of 171 signals during the entire study period, of which 99 (58%) were from women ( Table 5 ). Most signals ( *n* = 121; 71%; men: 75%; women: 68%) were generated during the spontaneous registration period.

**Table 5:** Signals ( *n* = 171) generated from the vigilance module, associated contacts/activities ( *n* = 75), and total number of medication adjustments

	Men	Women	Total
Signals, *n*	72	99	171
From spontaneous registrations	54	67	121
From predefined registrations	18	32	50
Contacts, *n*	23	52	75
Nurse contacts, *n* (% of contacts)	12 (52)	27 (52)	39 (52)
Nurse interventions	5 (22)	18 (35)	23 (31)
Other nurse contacts	7 (30)	9 (17)	16 (21)
Physician, contacts, *n* (% of contacts)	11 (48)	25 (48)	36 (48)
Medication reviews, with medication adjustments	2 (9)	7 (13)	9 (12)
Medication reviews, without any medication adjustment	2 (9)	4 (8)	6 (8)
Other physician contacts, with medication adjustments	6 (26)	8 (15)	14 (19)
Other physician contacts, without any medication adjustments	1 (4)	6 (12)	7 (9)
Medication adjustments, *n*	13	20	33

In total, 75 contacts were performed as a result of the signals from the VM, comprising a total of 23 patients, of whom a majority ( *n* = 15; 65%) were women. Thirty-one percent of the activities consisted of nurse interventions such as giving advice on prescribed medication, providing healthcare advice, measuring blood pressure, ECG, and applying compression stockings or bandages. In other cases, the contacts mainly consisted of booking a physician visit or preparing for a medication review.

Thirty-six (48%) of the 75 contacts were physician contacts, resulting in a total of 33 medication adjustments. In 15 of these contacts, a medication review was registered. In nine of these reviews, a total of 16 medication adjustments were made. Moreover, there were another 21 physician contacts not recorded as a medication review, of which 14 resulted in medication adjustments (in total 17 adjustments; Table 5 ). Sixty-one percent of all medication adjustments were made in women. The most common medication adjustments were dose adjustments or withdrawal of cardiac medications such as angiotensin II receptor blockers (six occasions), lipid-lowering agents (six occasions), diuretics (three occasions), calcium channel blockers (two occasions), and ACE inhibitors (one occasion). Out of the total of 33 medication adjustments performed, 14 (42%) remained at follow-up 3 months later.

## Discussion

### Main Findings

In this study, we have evaluated a model for the detection of potential ADEs, using an existing PCDSS with an additional VM, among home-dwelling patients. Our findings suggest a noticeable value of providing the possibility for patients to report symptoms at home, online to their primary care providers, creating the opportunity for prompt assessments and adjustments of their medication treatment, thus contributing to an increased medication safety. Forty-eight percent of our study population was subjected to medication related activities as a result of the signaling in the VM, generating a total of 33 medication adjustments among the 48 study participants.

### Drug Use

The drug use was extensive in our study population. The mean number of drugs was 8.6, and excessive polypharmacy (use of ≥10 drugs) 31 32 was seen in one-third of the cases. Use of drugs that are considered inappropriate for the elderly was uncommon (8%). However, potential DDIs were frequent. Moreover, the use of NSAIDs—which should be prescribed with caution to the elderly, mainly due to the risk of gastrointestinal bleeding and renal impairment—was noticeably high (17%) in comparison with another Swedish study, 33 which demonstrated a prevalence of six percent in a population aged 65 and older. This may partly be explained by the fact that many types of NSAIDs are available without a prescription and that these would be captured in this study, where the drug use was self-reported. This highlights the importance of gaining knowledge about the use of “over-the-counter” medicines in the older population.

### Symptoms

At baseline, the participants reported on average 4.7 symptoms—out of the 20 listed in the PHASE-20 form—creating moderate or major discomfort. Already, this indicated that some of the participants might have experienced ADE.

During the monitoring phase, the participants registered as many as 26 symptoms per person. The most frequently recorded symptoms were pain, tiredness, unsteadiness, and respiratory symptoms, which were also among the most reported at baseline. Tiredness, instability, and dizziness have been previously reported as symptoms associated with the use of cardiovascular agents, benzodiazepines, antidepressants, and opioid analgesics. 34 35 36 37 38

Pain and respiratory symptoms, on the other hand, are probably predominantly reflecting undertreatment rather than potential ADRs. The undertreatment of pain among older adults is a well-known problem. 39 40 41 42 Respiratory symptoms can be signs of insufficient pharmacological treatment of heart failure or chronic obstructive pulmonary disease. 43 44

### Gender Differences

The number of symptom registrations per patient were considerably higher in women (mean: 34) than in men (mean: 20). This is in line with a previous study showing a higher prevalence of reports of suspected ADRs among women than men. 45 Possible explanations of this marked gender difference may be either that women are more inclined to report medical problems to the healthcare, or that they in fact experience symptoms to a greater extent than men. 46

### Differences Between Spontaneously Reported and Predefined Symptoms

Seventy-one percent of the symptom registrations were made during the spontaneous registration period. Our primary hypothesis was that a patient provided with predefined alternatives would more easily associate and report a symptom as a possible ADE. However, the results show no support for this. One possible explanation for this is that our monitoring period lasted for 1 year, which is a relatively long period for the age group studied. This may have resulted in a certain level of monitoring fatigue and thus a reduced willingness to report. It is also possible that many of the symptoms recorded during the spontaneous period were resolved by then.

### Contacts/Activities

Forty-four percent of all signals generated by the VM resulted in contacts or activities at HCC. This response rate seems rather high; although it is difficult to compare, as similar studies, to our knowledge, have not been presented. In any case, this contrasts with the problems of low specificity and alert fatigue, which have been discussed as being major reasons for the low implementation rate of CDSS in healthcare. 47 48 49

One of the ideas with this study was that the study nurses should act as a “filter” for a possible physician contact/drug review. Fifty-two percent of all contacts related to the VM consisted of a nurse contact. The majority of these led to a booking of a physician visit or medication review, but one-third consisted of an independent nurse intervention, such as counseling, measuring blood pressure, ECG, or winding of the lower legs. Nurses have been reported to be particularly suited to detecting ADEs. 50 51 52 53 However, several studies also confirm nurses' uncertainty and inability to take that responsibility. Despite their pharmacological knowledge and wishes for them to be more active in pharmacovigilance work, including reporting of ADEs, their contribution to date has been reportedly limited. 54 55 56 57 Our study results suggest, however, that with the right tools and support, nurses can have an important role in monitoring and managing ADEs in older adults.

### Medication Reviews and Medication Adjustments

In 15 of the 36 physician contacts, a medication review was included, of which nine resulted in medication adjustments. In addition, medication adjustments were made in another 14 physician contacts without a medication review. The reason why not all medication adjustments were preceded by a complete medication review may be that it is time-consuming and therefore cannot always be performed, given the oftentimes high workload at HCC. Instead, the physician may focus on the current ADE and resolve it separately through a medication adjustment.

At any rate, nearly one-third of the contacts generated by the VM-signals resulted in medication adjustments. This speaks in favor of the need to involve the patients and retrieve information as close to the source as possible. Previous studies within the area of pharmacovigilance have also demonstrated the importance of patient participation and reporting of potential ADEs. 58 59 60

### Comparison with Other Studies on Computer-Aided Patient Self-Reporting

Despite numerous studies on the potential of a CDSS to improve medication safety, there is limited research evaluating a CDSS as a tool for self-reporting of symptoms associated with medication and how it prompts patients and caregivers to make appropriate medication changes, based on patient feedback. Lancaster and coworkers conducted a systematic review evaluating the impact of patients' use of eHealth tools. 61 Although they found little evidence for the effectiveness of such tools in improving medication recommendations and reconciliation, medication-use behavior, health service utilization, adverse effects, quality of life and patient satisfaction; they reported promising findings that specialized eHealth tools can be used for reporting and monitoring of symptoms and medication-related adverse effects, as well as evidence suggesting that use of eHealth tools may improve patient symptoms and lead to medication changes.

Further, Chrischilles and coworkers 62 demonstrated—using web-based personal health records—the possibility to engage older adults in medication self-management, potentially stimulating more medication reconciliation discussions with healthcare providers and increasing the patient's participation in medication safety.

To date, few studies have investigated methods to detect ADE in patients in their homes. One approach was presented by Schiff et al, 63 studying the impact of automated telephone calls coupled with phone-based pharmacist counselling, on the rate of detection of potential ADEs in patients newly prescribed medications for hypertension, diabetes, insomnia, and depression. The intervention led to a 50% increase in potential ADE symptoms documented, compared with matched controls. However, the response rate was very low; only one in eight patients in the overall intervention group participated in the call.

In another randomized, controlled trial, Weingart et al examined the use of a patient internet portal to prevent ADE. 64 A module within the portal sent a message asking the intervention patients if they had recently filled a prescription and whether they had experienced any problems with the medication. The patients' responses were automatically forwarded to primary care physicians. Half of the patients responded to at least one message. However, the results showed no significant impact on the rate of ADEs identified or on healthcare utilization.

What these studies have in common is that the communication was initiated by the healthcare providers, whereas in our study, the initiative came from the patients themselves, at a time and to an extent that was convenient for them. This may prove to be a more resource-effective approach.

### Limitations

This study has some limitations. A major one is the limited number of study participants. However, it is challenging to recruit participants in the relevant age group for a study with a digital monitoring approach, as the this one. As many as 30 percent of those screened had no access to a computer or tablet at all.

Another limitation was that we did not have a control group. The reason was that it would have been difficult to recruit enough study participants for two groups there and then. However, according to regional statistics, 19% of patients aged 75 or older listed in primary care in the region received a medication review during the studied period, of whom less than half were estimated to live in their own homes without home healthcare. Thus, with this comparison, at least three times more patients received a medication review in our study.

Further, our study does not include any data on user satisfaction. This is a major disadvantage as user satisfaction is considered a significant factor for the successful implementation of a CDSS. 65 Similarly, the usability or the technical stability and performance in the clinical environment were not examined. The original plan was to conduct qualitative focus group interviews with the users at the two HCCs, as well as interviews with a sample of the study patients, after completion of the 12-month study period. However, due to a lack of resources at the HCCs as well as the long study period and thus the patients' hesitancy to participate in the interviews, we had to omit this part of the study. This also meant that we could not explore the experiences of the two ways to report symptoms (spontaneous and predefined).

Finally, we cannot rule out some overestimation of activities associated with the alerts/signals. These data were extracted from the medical records, where the healthcare staff noted activities associated with a VM signal. We linked activities/contacts with the signals/alerts, if they were performed within a period of up to 10 days after the signal. On the other hand, it is also possible that an activity was sometimes initiated later than that and thus was not registered by us as being connected to a signal.

The increasing use of digital tools in society will likely lead to an increased willingness for studies and implementation of e-health tools among older adults. This, along with mobile phone applications becoming the solutions of choice, will accelerate the development of systems to facilitate communication between patients and healthcare providers. Such systems, for example, embedded in drug refill platforms, could also include support for involving patients in the follow-up of drug treatment and in drug reviews, thereby both supporting the doctor's work, empowering the patient, and strengthening their concordance.

## Conclusion

Our results suggest that patient participation by self-reporting symptoms via a PCDSS can contribute to safer drug use. To the best of our knowledge, models similar to this one, focusing on symptoms self-reported via a PCDSS, generating alerts that promote contacts with the healthcare providers for assessment of the medication treatment, have not been previously described in the literature. This could be a possible future way of working in primary healthcare, where the patients' “real-time” reporting of current symptoms can contribute to a more rapid assessment of their medications and the identification of ADEs.

## Clinical Relevance Statement

Our study shows that self-reporting of symptoms by elderly patients at home via a web-based CDSS can help detect ADEs, leading to medication adjustments and thus safer drug use.

## Multiple-Choice Questions

1. What proportion of adverse drug events in older people is estimated to be preventable?
  - One third
  - At least half
  - One in ten
  - One in four
  **Correct Answer** : The correct answer is option b. At least half of adverse drug events in older people are estimated to be preventable.
2. How many of the 36 physician contacts, resulting from signals from the vigilance module, led to medication adjustments?
  - 9
  - 15
  - 26
  - 23
  **Correct Answer** : The correct answer is option d. In 15 physician contacts, a medication review was included, of which nine resulted in medication adjustments. Additionally, medication adjustments were made in another 14 physician contacts without a medication review.
